# Molecular Dynamics Simulations of Polydopamine Nanosphere’s Structure Based on Experimental Evidence

**DOI:** 10.3390/polym14245486

**Published:** 2022-12-15

**Authors:** Jesús Manzanares-Gómez, Salvador León, Esteban Climent-Pascual, María Pilar García-Armada

**Affiliations:** 1 Departamento de Ingeniería Química y Medio Ambiente, Escuela Técnica Superior de Ingenieros Industriales, Universidad Politécnica de Madrid, José Gutierrez Abascal, 2, 28006 Madrid, Spain; 2 Nanomateriales 2D Para Aplicaciones en Energía y Sensores, Universidad Politécnica de Madrid, Unidad Asociada al CSIC, José Gutierrez Abascal, 2, 28006 Madrid, Spain

**Keywords:** polydopamine nanospheres, Au nanoparticles, molecular dynamics simulation

## Abstract

In this work, we show how to obtain internal monodispersed gold nanoparticles inside polydopamine (PDA) nanospheres that are also externally decorated with gold. The number of internal nanoparticles is affected by the size of the PDA nanosphere used, and the lower limit in the number of gold nanoparticles in the center of decorated nanospheres, one single gold nanoparticle, has been reached. In addition, extensive molecular dynamics simulations of PDA nanospheres based on four different chemical motifs, in the presence of water and with different sizes, have been performed to gain insight into the arrangements capable of accommodating cavities. In particular, PDA nanospheres based on pyranoacridinotrione (PYR) units provide good agreement with the experimental attainment of internal metal nanoparticles. In these, the stacking of PYR units leads to a particular morphology, with large portions of space occupied by the solvent, that would explain the observed formation of gold nanoparticles inside the PDA nanosphere.

## 1. Introduction

Polydopamine (PDA) is widely accepted as a biocompatible polymer and its applications generate increasing interest. Currently, it is possible to find more than 1222 scientific contributions related to “polydopamine” from only January to September 2022. The properties that attract so much attention are its biodegradability, ability to adhere to any material [1,2,3,4], high electrical conductivity [5], and interesting photothermal [6,7] and optical properties [8] (such as light absorption and dispersion). The presence of functional groups on its surface, such as amine, imine, or catechol [9,10] makes it possible to establish bonds with other molecules or biomolecules, or to chelate or coordinate metal ions for the formation of metal nanoparticles, e.g., gold nanoparticles (AuNPs), on their surface [11,12]. Due to these properties, PDA is used in a wide variety of applications, including pigments, solar screens, adhesives surface coverings, or core-shell composites [13,14]. More interestingly, under certain conditions, PDA nanospheres can be easily obtained [15] for promising applications such as drug transport and drug delivery systems [16,17,18], encapsulation of chemicals (such as pollutants for the removal of toxic compounds) [19], photovoltaics [20,21], and clinical [22,23,24,25], sensing [8,26,27], or even antibacterial applications [28]. From a chemical point of view, the great interest in PDA has two natural origins: on the one hand, from eumelanin (PDA), a pigment of the skin, hair, and brain [29,30,31]; on the other hand, from mussel adhesion proteins (mytilus foot protein) that adhere strongly to different substrates in an aqueous medium and are rich in 3,4-dihydroxy-L-phenylalanine, which derives from the neurotransmitter dopamine (DA) [18]. PDA can be easily and cheaply synthesized by air-oxidation of DA in aqueous weak-alkaline media onto the surface of almost all materials or it can be assembled as nanospheres. The PDA structure remains controversial and the polymerization mechanism is still discussed [32,33,34]. However, there is a consensus that the polymerization mechanism, kinetics, shape, and structure of PDA strongly depend on the mode of synthesis and/or the reaction conditions [7,35,36,37].

AuNPs are one of the more frequently used metal nanoparticles in a wide range of fields because of their excellent size-dependent properties and their overall chemical stability. As a study demonstrated, AuNPs are known to form on the surface of PDA nanospheres and microspheres by the coordination of chloroauric ions with the surficial functional groups of PDA and reduction by PDA catechol groups, or by adding a reductor, such as ascorbic acid [11,12]. In the resulting compounds, AuNPs were homogeneously distributed and anchored on the PDA surface, and no inner clusters or nanoparticles were detected in the TEM micrographs. Previously, the entry of chloroaurate ions into PDA shells grown around Fe_3_O_4_ nanoparticles was described [38]. In that report, it was shown that at low concentrations of HAuCl_4_, Au ions remain on the surface of the PDA shell, where the surface catechol groups reduce them to Au^0^. As the concentration of HAuCl_4_ increases, Au ions diffuse toward the interior of the PDA shell, where the reducing groups are more abundant, which makes the stabilizing interactions in the inner layer stronger than those on the surface. As a result, the formation of smaller and well-distributed AuNPs is observed. With higher concentrations, around 0.9 mM, the surface PDA groups are not sufficient and the excess of AuNP agglomerates, forming larger nanoparticles. In this case, the core of the nanoparticles was made up of magnetite, and the chloroaurate ions could not fully penetrate the PDA spheres. Other authors [39] also reported that AuNPs/PDA composites showed two different behaviors based on the concentration of the Au precursor in the absence of any added reductor. Synthesized AuNPs were homogeneously distributed at the surface of the PDA sphere’s surface when a sufficiently low concentration of Au precursor was used. However, if a high concentration of the Au precursor (10 mM) is used, a relatively smooth (or even unchanged) surface morphology is obtained, while an extremely high amount of little AuNPs is concentrated at the center of the sphere, occupying most of its inner space. So far, this unusual synthetic behavior has been attributed to the low density of the catechol groups and to the special interactions between the Au ions and the catechol groups. When a high concentration of Au ions accumulates in the center of the sphere, the functional groups or reducing agents reduce them, and as the reaction proceeds, the PDA spheres gradually fill with the AuNPs from the center to the edge. The synthesized AuNPs can then be released as core-shell AuPDA from between 5 and 45 nm by external stimuli, such as chemical treatment or NIR irradiation.

In recent years, new applications have been developed with hollow mesoporous polydopamine nanospheres of controlled dimensions, such as a carrier for drugs [40], photothermal synergetic therapy [41], or AuNPs-decorated PDA spheres as an electrochemical sensor of ascorbic acid, uric acid, dopamine, and tryptophan [42]. The latter allowed us to discover PDA nanospheres with small nanoparticles attached to the surface (6–7 nm) and cores constituted by discrete and monodisperse AuNPs of about 30–35 nm never before reported, grown inside PDA nanospheres. These developments are very innovative since, until now, these composites have required the prior synthesis of the monodisperse nanoparticles, and this procedure presents the difficulty of obtaining a homogeneous dispersion of the nanoparticles to avoid their agglomeration within the PDA.

Within the range of the conformational possibilities of PDA and taking into account the possibility of the functionalization of its surface, we consider it of great interest to continue studying the possibility of obtaining small and discrete AuNPs inside dopamine nanospheres, wherein the hollows and other parameters within the PDA spheres can control the number of the nanoparticles. As already mentioned, the shape and size of the PDA spheres can be controlled by the reaction conditions. It is known that the diameter of the PDA sphere decreases with the decreasing DA:NaOH molar ratio, and that the size and stability of PDA increase as the reaction time is increased [10,11]. In this way, it would be possible to obtain a single nanoparticle within the PDA nanosphere, and the composite would have important improved applications in, for example, surface-enhanced Raman scattering (SERS), therapy, and drug delivery.

**Figure 1:**
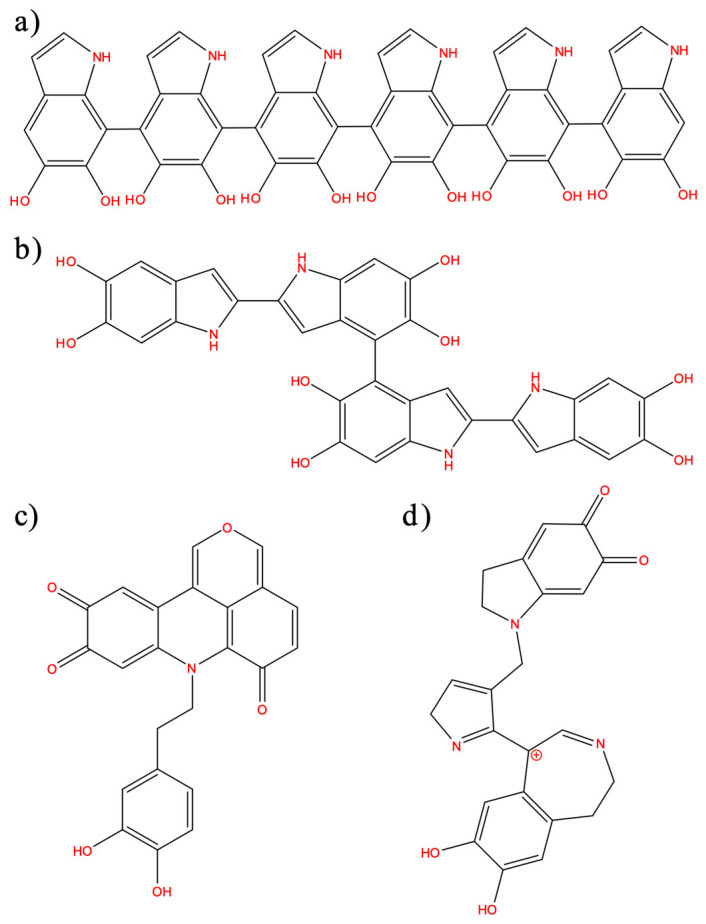
(**a**) Linear 5:6-dihydroxyindole (DHI)-based oligomers connected through 4,7 positions; (**b**) DHI-based oligomers with different combinations of connectivities at 2,4,7 positions; (**c**) oligomers based on a pyranoacridinotrione (PYR) motif; and (**d**) combination of a dopaminochrome unit, a 2H pyrrole moiety, and benzazepine (C_23_H_20_N_3_O_4_^+^, *m*/*z* 402).

The density and internal structure of PDA spheres depends on the synthesis method and are still controversial. To the best of our knowledge, the existence of internal voids or hollows and the entrance of ions from their external environment have not been studied yet. So far, the various structures proposed for PDA can be resumed as follows:

- Linear oligomers based on 5,6-dihydroxyindole (DHI) and 5,6-indolequinone connected through 4,7 positions (Figure 1a), confirmed by IR and UV-vis [10];
- DHI-based oligomers with different combinations of connectivities in 2,4,7 positions (Figure 1b), based on NMR studies on deuterated samples and mainly proposed by theoretical studies [43,44,45,46,47];
- Oligomers based on a pyranoacridinotrione (PYR) motif (Figure 1c), based on MALDI-MS and NMR [35,48];
- The combination of a dopaminochrome unit, a 2H-pyrrole moiety, and a benzazepine (chemical formula: C_23_H_20_N_3_O_4_^+^, *m*/*z* 402) (Figure 1d), based on MS studies using deuterium-labeled dopamine precursors [36].

The most recent experimental work [36] provided evidence that the major component of PDA is not DHI-related but dopaminochrome-related, and that porphyrin-like structures are not likely to be present in the PDA sequence, while PYR-based motifs appear to be a feasible candidate [48]. On the other hand, NMR studies [43] point out that the oligomers’ aggregation is promoted by π-π stacking interactions, and support the presence of DHI-based tetramers. In particular, these experimental data suggest that the phenyl/indole rings display low mobility, remaining rigid, because of the presence of these interactions. In addition, the same experiments also indicate that water diffusion is slow.

In summary, the starting points for this work are, firstly, the experimental evidence that it is possible to obtain discrete gold nanoparticles of controlled size inside a polydopamine sphere and, secondly, the lack of consensus on the polymer structure. The present work makes advances in the knowledge of the structure of polydopamine, with the knowledge that it is possible to obtain a certain number of discrete gold nanoparticles, even one single particle, within PDA nanospheres. This new approach deals with the description of their detailed structural properties as well as their possible relation with the formation of inner gold nanoparticles by molecular dynamics simulations. In order to account for the uncertainty in the chemical constitution, several models generated from different chemical candidates proposed in the literature [32,44,48] have been considered, including compounds based on DHI and on pyranoacridinotrione motifs. In addition, in the solution, models of different sizes have been simulated.

## 2. Materials and Methods

### 2.1. Materials

Dopamine hydrochloride, ascorbic acid, chloroauric acid, FeCl_2_·4H_2_O, and FeCl_3_·6H_2_O were purchased from Sigma-Aldrich (St. Louis, MO, USA). All other chemicals were used in analytical grade without further purifications. Ultrapure water was used for the preparation of all solutions.

### 2.2. Apparatus

Transmission electron microscopy (TEM) images were obtained by a JEOL JEM 2100 (Tokyo, Japan), operated at 200 kV (point resolution of 0.25 nm), and equipped with an X-ray energy dispersive spectroscopy (XEDS) analyzer. Scanning electron microscopy (SEM) images were obtained with a JEOL JSM 6400.

### 2.3. Synthesis of PDA Spheres

PDA spheres of several diameters (150 and 470 nm) were synthesized by procedures based on the previously described methods [42]. Briefly, the adequate quantity of NaOH solution to reach concentrations of 2.00 mM and 6.00 mM were added drop by drop to stirred 10.5 mM dopamine hydrochloride aqueous solutions. The mixture was maintained at 50 °C for 3 h while stirring, and a change of color from colorless, to pale yellow, and finally to dark brown was observed. The obtained PDA nanospheres were separated by centrifugation (12,000 rpm) and washed with ultrapure water several times. The PDNs were dried and stored at 4 °C. For later use, the spheres were re-dispersed in water by sonication.

### 2.4. Synthesis of Au-PDA Spheres

The re-dispersed PDA spheres were added into a 1 mM solution of HAuCl_4_, with the subsequent addition of 0.5 mM ascorbic acid as a reductor. The reaction was constantly stirred for 3 h at 30 °C. During this time, the color of the suspension turned gradually from dark brown to dark purple. The Au-PDA spheres were centrifuged and washed several times with ultrapure water and subsequently dried and stored in the dark at 4 °C [42].

### 2.5. Simulation Procedures

All the simulations were performed with the NAMD software [49] and the OPLS-AA force-field [50]. The TIP3P model was used for water molecules. Parameters for the simulations were obtained with the LigParGen web-based service [51,52,53]. The visualization and analysis of the results were performed with VMD [54], while PACKMOL [55] was used for the generation of bulk and solution-initial geometries.

The candidates considered for the PDA chemical constitution were the following:

- Linear 4,7-linked DHI-based octamers (lDHI), as shown in Figure 1a;
- DHI-based tetramers (TET-1 to TET-5) with diverging 2,4,7-connectivity, as shown in Figure 1b (the most stable candidates from DFT calculations were considered);
- Simple pyranoacridinetrione molecules (PYR), as shown in Figure 1c.

For each candidate, nanosphere models of two sizes were considered. Firstly, relatively small nanospheres of about 1 nm were generated in a box of water molecules; these systems consisted in 1024 repeat units for the DHI-like candidates (corresponding to 128 *l*DHI and 256 TET units, respectively), and 783 molecules for PYR. In addition, much larger models of about 20 nm were simulated, corresponding to 27,000 DHI-like units (3400 *l*DHI and 6800 TET monomers) and 10,000 PYR molecules. Due to the large size of these last systems, solvent effects were considered via the Generalized Born Implicit Solvent (GBIS) method [56]. While the size of the models is considerably smaller than those experimentally observed (whose dimensions are not practical for atomistic simulations), the analysis of the molecular dynamics can provide useful information on the overall behavior of these systems.

The following procedure for the generation of the PDA nanospheres in solution was adopted: low-density starting bulk (non-solvated) configurations were randomly generated with the PACKMOL software (São Paulo and Campinas, Brazil), and the structure was relaxed by a series of NVT short runs, each one with progressively smaller box dimensions, until the optimal density was achieved. The resulting configurations were then simulated in water solution (NVT ensemble for the systems with explicit water, after including the bulk PDA in a box of solvent; non-periodic constant temperature for the systems with implicit solvent). To avoid artifacts due to the particular starting configuration, the procedure was repeated up to 3 times for each model, using different randomly generated configurations. All the simulations were performed with a time step of 2 fs, and the duration of the production runs was 2.4 ns. A box of 17 × 17 × 17 nm was used for the systems with explicit solvent.

## 3. Results

**Figure 2:**
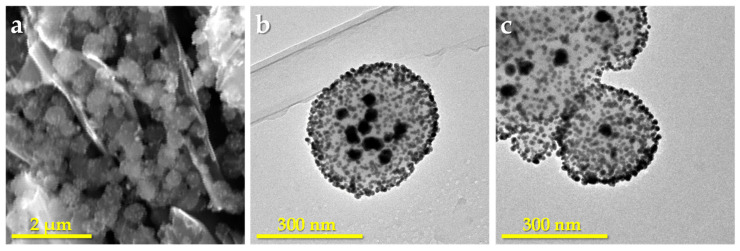
(**a**) SEM microphotograph of Au-PDA spheres of 470 nm on a graphite surface; (**b**) and (**c**) TEM micrographs of Au-PDA spheres of 470 nm and 150 nm, respectively.

In order to explore the possibility of obtaining different numbers of AuNPs inside PDA spheres, we prepared two sizes (150 and 470 nm) of PDA spheres by the same method used earlier [42]. Taking into account the dependence between the diffusion of the AuNP precursor inside the spheres and its concentration in the solution, as described in the introduction [38,39], and based on our own experience, we decided to work with a chloroaurate concentration of 1 mM and to use ascorbic acid as an added reducing agent. Figure 2 shows the Au-PDA spheres obtained with the two sizes of PDA. As can be seen, Figure 2b,c show both types of gold nanoparticles, the outer smaller ones and the inner ones of about 30–35 nm diameter. As previously observed, the smaller Au-PDA nanospheres are less stable than the larger ones and some appear aggregated. However, it can be observed how a single 30–35 nm AuNP has formed in the center of each sphere. It can also be observed that the size of the AuNPs are similar regardless of the size of the PDA sphere and, unlike what has been observed by other authors, there is no accumulation of smaller AuNPs inside [39]. The inner larger nanoparticles are not visible in SEM micrographs, confirming that they were generated inside the spheres (Figure 2a).

The shape and size of AuNPs have also been related with the organic reductor used to obtain them [57,58]. In fact, it was observed that when glucose, sucrose, and fructose are used as reductors for the synthesis of AuNPs, the strongest reductors, such as glucose or sucrose, generate particles too small for colloidal stability and therefore tend to coalescence, producing NPs of variable size. However, fructose allows the obtaining of uniform larger NPs [59]. In our case, a 1 mM chloroaurate solution was used, and the obtained AuNPs anchored to the surface of the PDA spheres were about 7 nm in diameter when ascorbic acid was used as the reductor agent. According to the above, the presence of the additional reductor agent, such as ascorbic acid, prevents the agglomeration of the AuNPs and, therefore, the nanoparticles formed on the surface are small despite the high concentration of HAuCl_4_. On the other hand, the 30–35 nm AuNPs observed within the PDA nanospheres must be attributed to the diffusion of chloroauric acid in the core of the PDA nanosphere, which cannot be accessed by ascorbic acid, probably by steric hindrance. The fact that these internal particles are the same size and one or more are formed depending on the size of the sphere can be attributed to the hollows of the structure of each PDA sphere and the distribution of the functional groups within them.

In our opinion, the discovery of the existence of these isolated nanoparticles within the PDA nanospheres could expand the field of applications of these composites due to the possibility of controlling their quantity and size, which requires knowledge of the internal structures of nanospheres.

**Figure 3:**
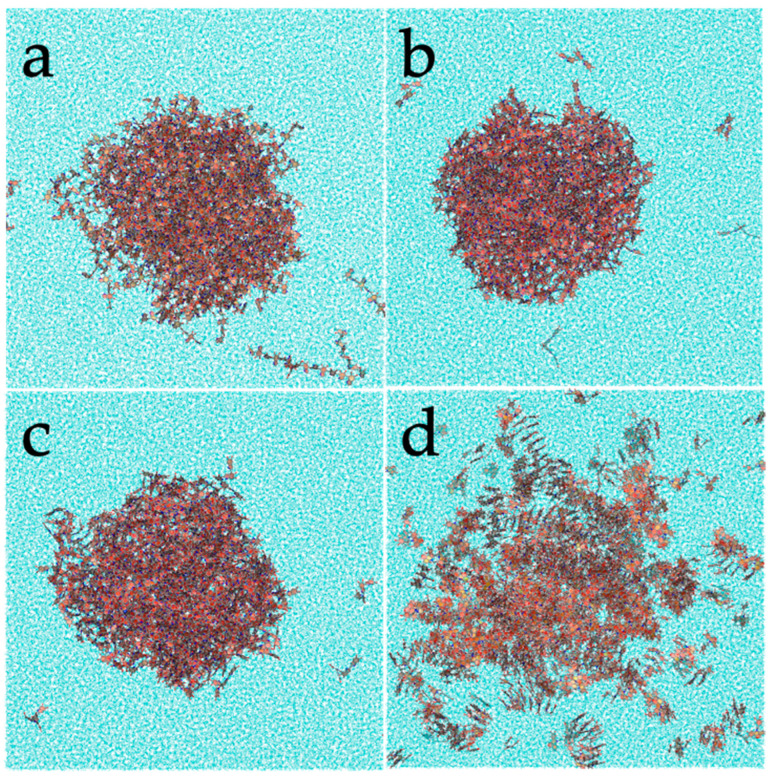
Representation of one configuration for the different PDA models: (**a**) *l*DHI, (**b**) TET-1, (**c**) TET-2, and (**d**) PYR, in solution from the MD simulations.

Taking into account the above-mentioned observations, extensive molecular dynamics simulations were performed. Due to the uncertainty regarding the chemical motif of PDA, four candidates were considered. The morphology displayed by the smaller models in the molecular dynamics’ trajectories with explicit solvent is shown in Figure 3. While the three DHI-based models (*l*DHI, TET-1, and TET-2) exhibit a similar morphology, with a rather spherical compact aggregation of molecules, the PYR model presents a clearly different geometry. More specifically, molecules are stacked, and arranged in disordered vermicular groups with a larger degree of dispersion than those observed in the DHI-based models, to form a rather starfish-like distribution. Differences also appear in the dimensions of the particles: for instance, the average radius of gyration for the PYR nanospheres is about 6.10 nm, much larger than the values for the other models (3.80 for *l*DHI, 3.70 for TET-1, and 3.80 for TET-2). In all cases, the three components of the radius of gyration had almost the same values, indicating that the aggregates have a low degree of anisotropy or asphericity.

**Figure 4:**
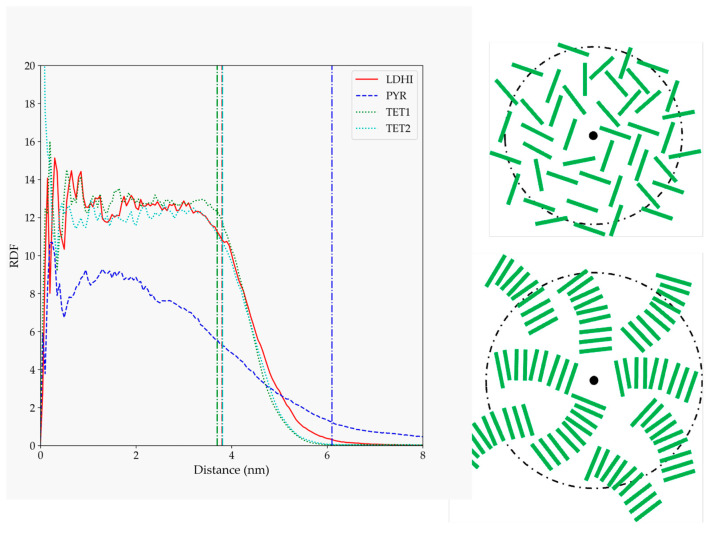
RDF for PDA atoms relative to the position of the nanosphere center of mass for the four models considered in solution (*l*DHI: red; PYR: blue; TET-1: green; TET-2: cyan). All the profiles are averaged for the three configurations generated for each model. The vertical lines indicate the average approximate value of the radius of gyration for each of the systems. The schemes on the right are simplified depictions proposed for *l*DHI, TET-1, and TET-2 (**top**), and for PYR (**bottom**) from the overall geometry and the RDF profiles.

The details concerning the arrangement of the oligomers within the PDA aggregates were analyzed in terms of their radial distribution function relative to the center of the nanosphere (Figure 4). Again, the three DHI-based models show a similar behavior, with a rather homogeneous profile (a relatively uniform density of oligomers) that spans for about the average radius of gyration (indicated by vertical lines in Figure 4), and then decreases up to 1–2 nm. For PYR, on the other hand, the starfish-like arrangement leads to a distribution profile that, at short distances from the center, starts to slowly decrease for an extended length, thus indicating a lower density of oligomers in the interior of the nanosphere. These differences observed in the profiles are illustrated in the right panel of Figure 4, with simplified depictions of the oligomers’ distributions based on Figure 3.

Experimental studies based on NMR techniques [43] have ascribed the aggregation of the PDA monomers-oligomers to π-π interactions related with a rigid planar conformation of the individual units. In this work, the planarity of the different candidate molecules in each model of PDA nanospheres was analyzed in terms of two metrics of molecular planarity recently proposed, namely the molecular planarity parameter (MPP) and the span of deviation from plane (SDP) [60]. The first metric (MPP) is computed as the root-mean-square deviation of the atoms from a plane fitted to the molecule, and the second parameter (SDP) as the difference between the squares of the distance to the plane for the two atoms set further apart from both sides of the plane. Hence, these two definitions provide a metric on the deviation of a molecule from a completely planar geometry, where small values for both parameters are indicative of a planar system.

**Figure 5:**
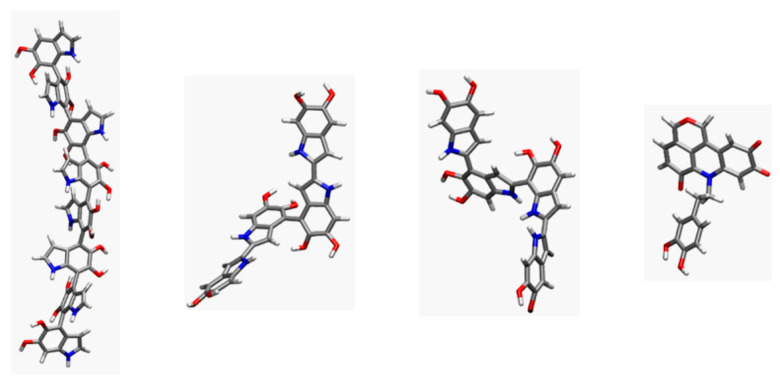
Examples of representative conformations from the molecular dynamics trajectories for individual units for the four PDA models considered, in the MD simulations with explicit solvent (left to right: *l*DHI, TET-1, TET-2, and PYR).

The values reported in Table 1 indicate both that PYR displays the smallest deviations (in both parameters), that is, the largest degree of planarity, and that the TET-1 and TET-2 tetramers, and especially the *l*HDI octamer, exhibit a remarkable deviation from planarity. This behavior is illustrated in Figure 5, where the geometry of individual molecules taken from the simulations for the four candidates is displayed. Inspection of the corresponding torsions shows that the bonds connecting different DHI units are neither rigid nor in a planar conformation.

**Figure 6:**
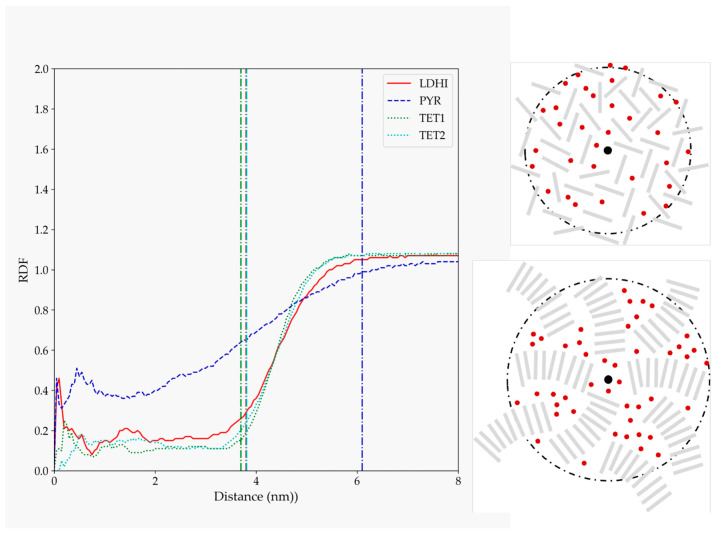
RDF for water atoms relative to the position of the nanosphere center of mass for the four models considered in explicit solvent (*l*DHI: red; PYR: blue; TET-1: green; TET-2: cyan). All the profiles are averaged for the three configurations generated for each model. The vertical lines indicate the average approximate value of the radius of gyration for each of the systems. The schemes on the right are simplified depictions for the water penetration proposed for *l*DHI, TET-1, and TET-2 (**top**), and for PYR (**bottom**) from the overall geometry and the RDF profiles.

The possible implications of the conformational and morphological properties of the different aggregates on the formation of AuNPs in their interior were considered. As stated in the introduction, experimental evidence points to a slow diffusion of water molecules inside the PDA nanospheres, which, in the presence of aurate salts, could explain the presence of solved gold-containing molecules. These could eventually produce encapsulated AuNPs upon reduction. Therefore, the distribution of water molecules inside the different aggregates was analyzed in terms of the radial distribution function relative to the distance to the center of the nanosphere, as shown in Figure 6.

**Figure 7:**
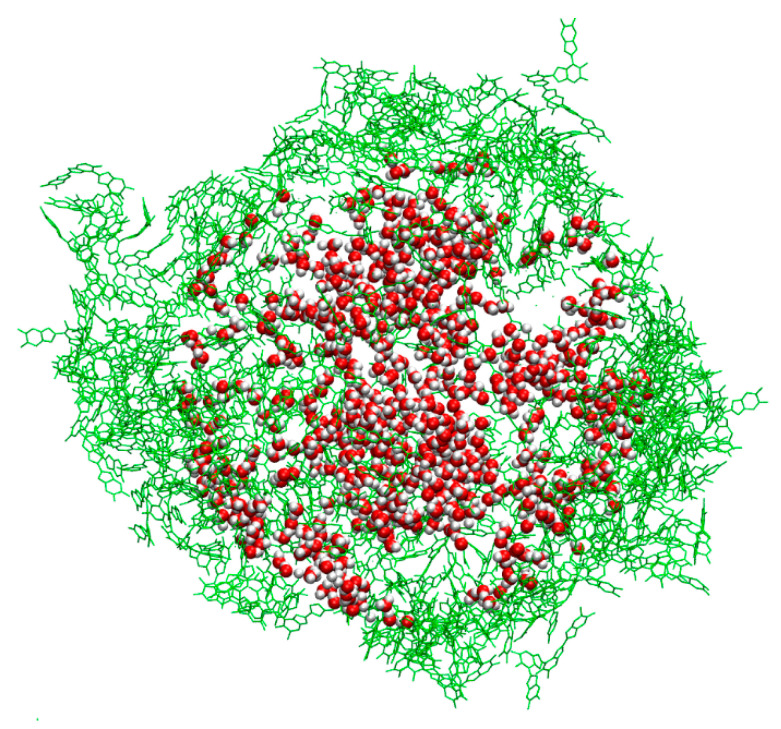
Distribution of water molecules within a PDA nanosphere in the TET-2 model. In green are displayed the outer TET-2 oligomers to indicate the dimensions of the nanosphere.

The profiles displayed for the four models indicate that there is a non-negligible insertion of water inside all the systems. In particular, the density of water inside for the DHI-based models is rather low, with a uniform profile clearly located within the nanosphere. On the other hand, the presence of water inside the PYR cluster is larger, and the profile does not display a large change between the interior and exterior of the nanosphere. A schematic depiction of these behaviors is included on the right side of Figure 6. To further illustrate these trends, Figure 7 shows the distribution of water molecules within an aggregate based on the TET-2 candidate. Obviously, the starfish-like morphology of the PYR system allows for the presence of a much larger amount of water within the aggregate, but it should be taken into account that, in contrast to the DHI-based models, those molecules are not confined in a rather closed geometry. Thus, a large diffusion of water should be expected.

**Figure 8:**
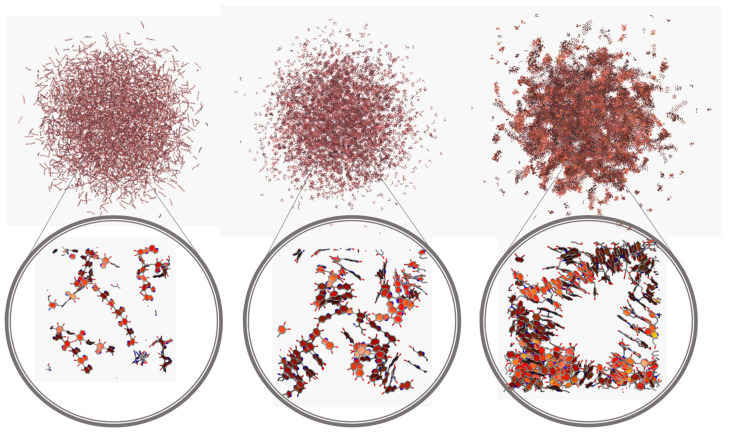
Representation of one configuration for the different PDA models (left to right: *l*DHI, TET-1, and PYR) in implicit solvent from the MD simulations.

To gain more insight into the behavior of the different models, additional MD simulations were carried out considering much larger nanospheres, up to about 20 nm. Due to the huge number of atoms involved, solvent effects were incorporated with an implicit model, namely GBIS [56]. On the other hand, as the behavior observed thus far for both TET-1 and TET-2 was very similar, only a system constituted by TET-1 was considered. Snapshots of the corresponding simulations are shown in Figure 8. It can be seen that, for larger nanospheres, some of the general trends observed in the smaller systems become more apparent. In particular, while *l*DHI and TET still display a rather homogenous distribution of molecules, the extensive stacking of PYR molecules leads to a morphology with the presence of big inner cavities.

The geometry of the large nanospheres was characterized in terms of the relative shape anisotropy (κ^2^) derived from the principal moments of the gyration tensor. The average values obtained for the three systems were very low (see Table 2), of the order of 10^−4^, indicative of geometries with a uniform, spherical shape.

**Figure 9:**
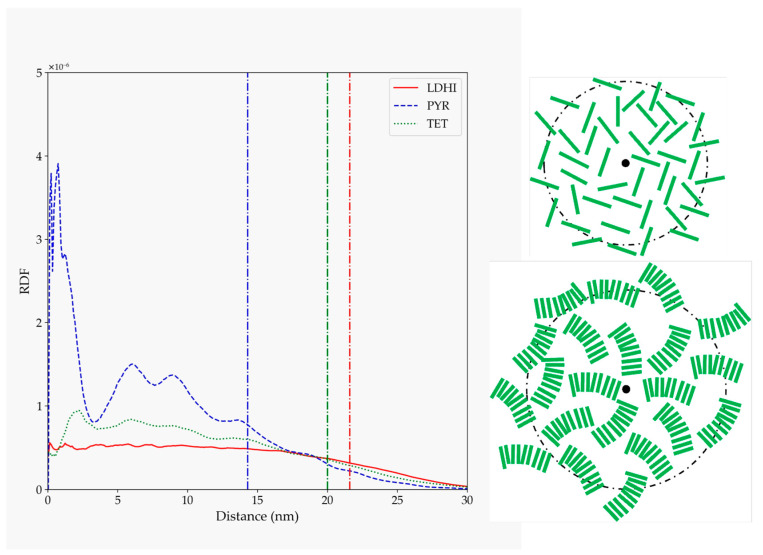
Radial distribution function of the rings of different monomer units (referred to their centers of mass) relative to the nanosphere center of mass for the large nanosphere models (*l*DHI: red; PYR: blue; TET: green), in the simulations with implicit solvent. The vertical lines indicate the average approximate value of the radius of gyration for each of the systems. The schemes on the right are simplified depictions proposed for *l*DHI and TET-1 (**top**), and for PYR (**bottom**) from the overall geometry and the RDF profiles.

The morphology differences become more evident in the distribution of the molecules with respect to the center of the nanospheres, as shown in Figure 9. The two DHI-based models display a uniform (disordered) distribution of units in the whole nanosphere, as in the one schematically depicted in the upper right corner of Figure 9. On the other hand, the function for the PYR model exhibits clear localized peaks in the inner core of the nanosphere, indicative of a structured arrangement close to the center that becomes more dispersed at larger distances. Taking as a reference the geometry displayed in Figure 8, this profile may be interpreted in terms of a distribution like that shown in the lower right corner of Figure 9.

**Figure 10:**
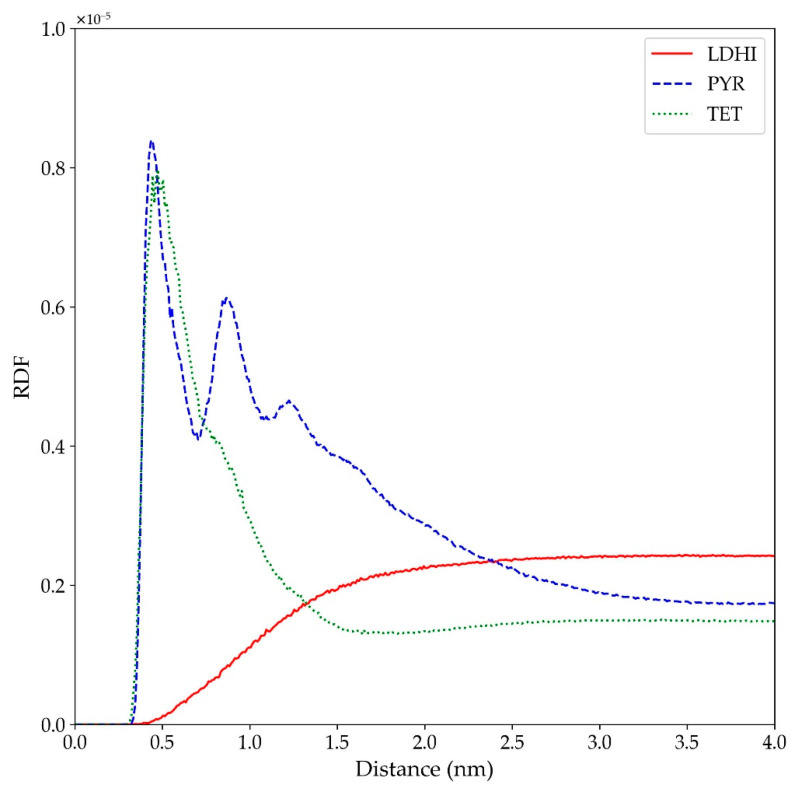
Radial distribution function among the rings of different monomer units (referred to their ring centers in each unit) for the large nanosphere models (lDHI: red; PYR: blue; TET: green) in implicit solvent.

In a similar fashion as with the simulations for the smaller nanospheres, the occurrence of π-π stacking interactions in the larger models was investigated to get a better comparison with some of the experimental evidence. In particular, the stacking between units in the larger nanospheres was analyzed in terms of the pair-pair radial distribution function, computed in terms of the centers of the rings in each unit. The results are displayed in Figure 10 and reveal an extensive stacking between different molecules for the PYR model, covering up to 3 to 4 stacked units (as shown by the peaks in the corresponding distribution). On the other hand, the degree of stacking is much smaller for TET, mainly up to two units, while for *l*DHI it is practically non-existent. Therefore, it can be said that the PYR-based model is the one that displays a more planar conformation. More importantly, it is the one that exhibits a larger degree of aggregation with π-π interactions and, therefore, better agrees with NMR-based observations [43].

**Figure 11:**
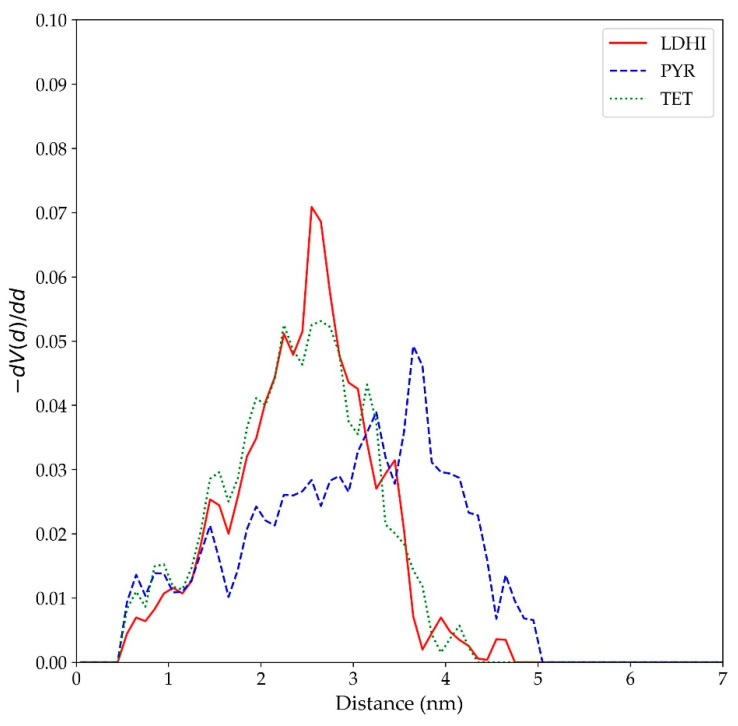
Pore size distributions for the large nanosphere models (lDHI: red; PYR: blue; TET: green) in implicit solvent. *V(d)* refers to the accessible volume of a cavity (“pore”) of a diameter *d*.

Indeed, the extensive stacking in the PYR-based model appears to play an important role in the general features of the nanosphere, specifically in the degree of water penetration within the aggregate. As illustrated in the morphologies in both Figure 3 and Figure 8, the π-π interactions in PYR favors the formation of vermicular aggregates that assemble together to form a nanosphere with large portions of empty space that can be occupied by water. Such an arrangement is clearly different from the DHI-based models, which exhibit a uniform distribution of units. The structural details of these free space (or cavities) have been characterized in terms of a pore size distribution (PSD) analysis, using the PoreBlazer v4.0 software [61]. In this method, which has proven useful for the analysis of polymer systems [62,63], a given configuration is mapped to a lattice grid, and a hard-sphere probe is used to locate and measure free spaces (cavities or “pores”). In this study, the PSD analysis was carried out for the inner region (a cube with a side length of 20 nm) in the final configuration of each large nanosphere model. The results, shown in Figure 11, indicate that the most probable free space size for *l*DHI and TET is about 2.5 nm in diameter, while cavities with a maximum of about 4 nm were observed for the PYR nanosphere (right side of Figure 8).

Overall, a remarkable diffusion of solvated molecules, as chloroaurate ions, inside the nanosphere can be expected for the PYR-based model, given its morphological features. In other words, the geometry displayed by the PYR-based model could account for both the occurrence of π-π stacking as suggested by NMR experiments, and the formation of gold nanoparticles inside PDA nanospheres.

## 4. Conclusions

This work confirmed the preparation of discrete and monodispersed gold nanoparticles of about 30–35 nm inside gold-decorated polydopamine spheres. Under fixed preparation conditions, the number of internal gold nanoparticles is affected by the PDA nanosphere size, and the lower limit of a single gold nanoparticle in the center of the decorated nanospheres was reached with 150 nm nanospheres.

Likewise, this work provides a new contribution to the knowledge of the structure of PDA spheres and their interior space through extensive molecular dynamics simulations. For this purpose, MD simulations of PDA nanospheres with two different sizes, based on four different chemical motifs (lDHI, TET-1, TET-2, and PYR), in the presence of water, were performed with the aim of gaining insight into the arrangements capable of accommodating cavities that could explain the formation of the internal nanoparticles. Overall, the results of the MD simulations, especially when considering systems of a larger size, suggest that the pyranoacridinotrione-based model displays features that better agree with experimental observations. On the one hand, the π-π interactions in PYR favor the formation of vermicular aggregates that assemble together to form a nanosphere, allowing large portions of empty space that can be occupied by water. Furthermore, the occurrence of these π-π interactions that extend over several molecules is compatible with the evidence attained from NMR studies. On the other hand, regarding the DHI-based models, the relative lack of planarity, and hence rather small presence of stacking between molecules, leads to rather uniform nanospheres that are not supported by experimental observations. Consequently, it was demonstrated that MD simulations are a good tool to providing a better understanding of the structure of polydopamine nanospheres and to explaining the formation of internal AuNPs, agreeing with the SEM and previous NMR experimental evidence.

## Figures and Tables

**Table 1:** Values obtained for the planarity parameters (MPP and SDP), averaged for the oligomeric units for the three MD trajectories in solution for the nanosphere models for the four candidates considered for PDA.

Model	MPP	SDP
*l*DHI	6.12	21.78
TET-1	3.20	12.05
TET-2	3.04	11.55
PYR	2.34	8.39

**Table 2:** Relative shape anisotropy (κ^2^) from the MD trajectories in implicit solvent for the large nanosphere models for the three candidates considered for PDA.

Model	κ^2^
*l*DHI	2.5·10^−4^
TET-1	4.5·10^−4^
PYR	3.2·10^−4^

## Data Availability

The raw and processed data required to reproduce these findings cannot be shared at this time due to technical or time limitations.

## References

[1] Li X., Zhu J., Zhang B., Jiao Y., Huang J., Wang F. (2021). Manganese dioxide nanosheets decorated on MXene (Ti_3_C_2_T_x_) with enhanced performance for asymmetric supercapacitors. Ceram. Int..

[2] Mei H., Gao Z., Wang Q., Sun H., Zhao K., Zhang P., Hao J., Ashokkumar M., Cui J. (2021). Ultrasound expands the versatility of polydopamine coatings. Ultrason. Sonochem..

[3] Alfieri M.L., Weil T., Wah D.Y., Ball V. (2022). Polydopamine at biological interfaces. Adv. Colloid Interface Sci..

[4] Feinberg H., Hanks T.W. (2022). Polydopamine: A bioinspired adhesive and surface modification platform. Polym. Int..

[5] Eom T., Lee J., Lee S., Ozlu B., Kim S., Martin D.C., Shim B.S. (2022). Highly Conductive Polydopamine Coatings by Direct Electrochemical Synthesis on Au. ACS Appl. Polym. Mater..

[6] Qi X., Pan W., Tong X., Gao T., Xiang Y., You S., Mao R., Chi J., Hu R., Zhang W. (2021). ε-Polylysine-stabilized agarose/polydopamine hydrogel dressings with robust photothermal property for wound healing. Carbohydr. Polym..

[7] Du Y., Huang H., Hu X., Liu S., Sheng X., Li X., Lu X., Qu J. (2021). Melamine foam/polyethylene glycol composite phase change material synergistically modified by polydopamine/MXene with enhanced solar-to-thermal conversion. Renew. Energy.

[8] Zhu X., Yan B., Yan X., Wei T., Yao H., Mia M.S., Xing T., Chen G. (2020). Fabrication of non-iridescent structural color on silk surface by rapid polymerization of dopamine. Prog. Org. Coat..

[9] Khan Z., Shanker R., Um D., Jaiswal A., Ko H., Khan A., Jawaid M., Parwaz Khan A.A., Asiri A.M. (2018). Bioinspired Polydopamine and Composites for Biomedical Applications. Electrically Conductive Polymers and Polymer Composites: From Synthesis to Biomedical Applications.

[10] Wu M., Wanga T., Müllerb L., Müller F.A. (2020). Adjustable synthesis of polydopamine nanospheres and their nucleation and growth. Colloids Surf. A.

[11] Liu X., Zhang X.Y., Wang L.L., Wang Y.Y. (2014). A Sensitive Electrochemical Sensor for Paracetamol Based on a glassy carbon electrode modified with Multiwalled Carbon Nanotubes and Dopamine Nanospheres Functionalized with Gold Nanoparticles. Microchim. Acta.

[12] Han X., Chen X., Yan M., Liu H. (2019). Synergetic effect of polydopamine particles and in-situ fabricated gold nanoparticles on charge-dependent catalytic behaviours. Particuology.

[13] Xu M., Yan L., Zhu Y., Li Y., Song X., Yin L. (2020). Polydopamine-coated gold nanoparticles used as modifier of the electron transport layer for TB7:PC71BM polymer solar cells. J. Mater. Sci. Mater. Electron..

[14] Zhao N., Liu S., Xing J., Pi Z., Song F., Liu Z. (2020). Trace determination and characterization of ginsenosides in rat plasma through magnetic dispersive solid-phase extraction based on core-shell polydopamine-coated magnetic nanoparticles. J. Pharm. Anal..

[15] Ju K., Lee Y., Lee S., Park S.B., Lee J. (2011). Bioinspired Polymerization of Dopamine to Generate Melanin-Like Nanoparticles Having an Excellent Free-Radical-Scavenging Property. Biomacromolecules.

[16] Liu R., Guo Y., Odusote G., Qu F., Priestley R.D. (2013). Core–Shell Fe_3_O_4_ Polydopamine Nanoparticles Serve Multipurpose as Drug Carrier, Catalyst Support and Carbon Adsorbent. ACS Appl. Mater. Interfaces.

[17] Oroujeni M., Kaboudin B., Xia W., Jöhsson P., Ossipov D.A. (2018). Conjugation of Cyclodextrin to Magnetic Fe_3_O_4_ Nanoparticles via Polydopamine Coating for Drug Delivery. Prog. Org. Coat..

[18] Li H., Yin D., Li W., Tang Q., Zou L., Peng Q. (2021). Polydopamine-based nanomaterials and their potentials in advanced drug delivery and therapy. Colloids Surf. B Biointerfaces.

[19] Dong Z., Gong H., Gao M., Zhu W., Sun X., Feng L., Fu T., Li Y., Liu Z. (2016). Polydopamine Nanoparticles as a Versatile Molecular Loading Platform to Enable Imaging-guided Cancer Combination Therapy. Theranostics.

[20] Antidormi A., Melis C., Canadell E., Colombo L. (2017). Assessing the Performance of Eumelanin/Si Interface for Photovoltaic Applications. J. Phys. Chem. C.

[21] Pinna E., Melis C., Antidormi A., Cardia R., Sechi E., Cappellini G., d’Ischia M., Colombo L., Mula G. (2017). Deciphering Molecular Mechanisms of Interface Buildup and Stability in Porous Si/Eumelanin Hybrids. Int. J. Mol. Sci..

[22] Zeng Q., Qian Y., Huang Y., Ding F., Qi X., Shen J. (2021). Polydopamine nanoparticle-dotted food gum hydrogel with excellent antibacterial activity and rapid shape adaptability for accelerated bacteria-infected wound healing. Bioact. Mater..

[23] Afrash H., Nazeri N., Davoudi P., Majidi R.F., Ghanbari H. (2021). Development of a Bioactive Scaffold based on NGF Containing PCL/Chitosan Nanofibers for Nerve Regeneration. Biointerface Res. Appl. Chem..

[24] Yazdi M.K., Zare M., Khodadadi A., Seidi F., Sajadi S.M., Zarrintaj P., Arefi A., Saeb M.R., Mozafari M. (2022). Polydopamine Biomaterials for Skin Regeneration. ACS Biomater. Sci. Eng..

[25] Chinchulkar S.A., Patra P., Dehariya D., Yu A., Rengan A.K. (2022). Polydopamine nanocomposites and their biomedical applications: A review. Polym. Adv. Technol..

[26] Guo J., Liu D., Yang Z., Wenge W., Chan E.W.C., Zeng Z., Wong K.Y., Lin P., Chen S. (2020). A photoelectrochemical biosensor for rapid and ultrasensitive norovirus detection. Bioelectrochemistry.

[27] Szewczyk J., Aguilar-Ferrer D., Coy E. (2022). Polydopamine films: Electrochemical growth and sensing applications. Eur. Polym. J..

[28] Xu Z., Wang T., Liu J. (2022). Recent Development of Polydopamine Anti-Bacterial Nanomaterials. Int. J. Mol. Sci..

[29] Ito S., Wakamatsu K., d’Ischia M., Napolitano A., Pezella A., Borovanský J., Riley P.A. (2011). Structure of Melanins. Melanins and Melanosomes: Biosynthesis, Biogenesis, Physiological, and Pathological Functions.

[30] d’Ischia M., Napolitano A., Pezella A., Meredith P., Sarna T. (2009). Chemical and structural diversity in eumelanins: Unexplored bio-optoelectronic materials. Angew. Chem. Int. Ed..

[31] Panzella L., Gentile G., D’Errico G., Della Vecchia N.F., Errico M.E., Napolitano A., Carfagna C., d’Ischia M. (2013). Atypical structural and π-electron features of a melanin polymer that lead to superior free-radical-scavenging properties. Angew. Chem. Int. Ed..

[32] Liebscher J., Mrówczyński R., Scheidt H.A., Filip C., Hădade N.D., Turcu R., Bende A., Beck S. (2013). Structure of Polydopamine: A Never-Ending Story?. Langmuir.

[33] d’Ischia M., Napolitano A., Ball V., Chen C.T., Buehler M.J. (2014). Polydopamine and eumelanin: From structure-property relationships to a unified tailoring strategy. Acc. Chem. Res..

[34] Lin S., Chen C.T., Bdikin I., Ball V., Grácio J., Buehler M.J. (2014). Tuning heterogenous poly(dopamine) structure and mechanics: In silico covalent cross-linking and thin film nanoindentation. Soft Matter.

[35] Liebscher J. (2019). Chemistry of Polydopamine—Scope, Variation and Limitation. Eur. J. Org. Chem..

[36] Lyu Q., Hsueh N., Chai C.L.L. (2019). Unravelling the polydopamine mystery: Is the end in sight?. Polym. Chem..

[37] Cho S., Kim S.H. (2015). Hydroxide ion-mediated synthesis of monodisperse dopamine-melanin nanospheres. J. Colloid Interface Sci..

[38] Zeng T., Zhang X.L., Niu H.Y., Ma Y.R., Li W.H., Cai Y.Q. (2013). In situ growth of gold nanoparticles onto polydopamine-encapsulated magnetic microspheres for catalytic reduction of nitrobenzene. Appl. Catal. B Environ..

[39] Lee Y.S., Bae J.Y., Koo H.Y., Lee Y.B., Cho W.S. (2016). A remote-controlled generation of gold@polydopamine (core@shell) nanoparticles via physical-chemical stimuli of polydopamine/gold composites. Sci. Rep..

[40] Lin K., Gan Y., Zhu P., Li S., Lin C., Yu S., Zhao S., Shi J., Li R., Yuan J. (2021). Hollow mesoporous polydopamine nanospheres: Synthesis, biocompatibility and drug delivery. Nanotechnology.

[41] Li S., Gan Y., Lin C., Lin K., Hu P., Liu L., Yu S., Zhao S., Shi J. (2021). NIR-/pH-Responsive Nanocarriers Based on Mesoporous Hollow Polydopamine for Codelivery of Hydrophilic/Hydrophobic Drugs and Photothermal Synergetic Therapy. ACS Appl. Bio Mater..

[42] Arroquia A., Acosta I., García Armada M.P. (2020). Self-assembled gold decorated polydopamine nanospheres as electrochemical sensor for simultaneous determination of ascorbic acid, dopamine, uric acid and tryptophan. Mater. Sci. Eng. C.

[43] Cîrcu M., Filip C. (2018). Closer to the polydopamine structure: New insights from a combined ^13^C/^1^H/^2^H solid-state NMR study on deuterated samples. Polym. Chem..

[44] Chen C.-T., Martin-Martinez F.J., Seob Jung G., Buehler M.J. (2017). Polydopamine and eumelanin molecular structures investigated with ab initio calculations. Chem. Sci..

[45] Dreyer D.R., Miller D.J., Freeman B.D., Paul D.R., Bielawski C.W. (2012). Elucidating the structure of poly(dopamine). Langmuir.

[46] Cheng J., Moss S.C., Eisner M., Zschack P. (1994). X-Ray characterization of melanins I. Pigm. Cell. Res..

[47] Chen C.-T., Chuang C., Cao J., Ball V., Ruch D., Buehler M.J. (2014). Excitonic effects from geometric order and disorder explain broadband optical absorption in eumelanin. Nat. Commun..

[48] Alfieri M.L., Micillo R., Panzella L., Crescenzi O., Oscurato S.L., Maddalena P., Napolitano A., Ball V., d’Ischia M. (2018). Structural Basis of Polydopamine Film Formation: Probing 5,6-Dihydroxyindole-Based Eumelanin Type Units and the Porphyrin Issue. ACS Appl. Mater. Interfaces.

[49] Phillips J.C., Braun R., Wang W., Gumbart J., Tajkhorshid E., Villa E., Chipot C., Skeel R.D., Kale L., Schulte K. (2005). Scalable molecular dynamics with NAMD. J. Comput. Chem..

[50] Jorgensen W.L., Tirado-Rives J. (1988). The OPLS [optimized potentials for liquid simulations] potential functions for proteins, energy minimizations for crystals of cyclic peptides and crambin. J. Am. Chem. Soc..

[51] Jorgensen W.L., Tirado-Rives J. (2005). Potential energy functions for atomic-level simulations of water and organic and biomolecular systems. Proc. Natl. Acad. Sci. USA.

[52] Dodda L.S., Vilseck J.Z., Tirado-Rives J., Jorgensen W.L. (2017). 1.14*CM1A-LBCC: Localized Bond-Charge Corrected CM1A Charges for Condensed-Phase Simulations. J. Phys. Chem. B.

[53] Dodda L.S., Cabeza de Vaca I., Tirado-Rives J., Jorgensen W.L. (2017). LigParGen web server: An automatic OPLS-AA parameter generator for organic ligands. Nucleic Acids Res..

[54] Humphrey W., Dalke A., Schulten K. (1996). VMD—Visual Molecular Dynamics. J. Mol. Graph..

[55] Martínez L., Andrade R., Birgin E.G., Martínez J.M. (2009). Packmol: A package for building initial configurations for molecular dynamics simulations. J. Comput. Chem..

[56] Qiu D., Shenkin P.S., Hollinger F.P., Still W.C. (1997). The GB/SA continuum model for solvation. A fast analytical method for the calculation of approximate Born radii. J. Phys. Chem..

[57] Baruwati B., Varma R.S. (2009). High Value Products from Waste: Grape Pomace Extract—A Three-in-One Package for the Synthesis of Metal Nanoparticles. ChemSusChem.

[58] Zhao Y., Yeh Y., Liu R., You J., Qu F. (2015). Facile deposition of gold nanoparticles on core-shell Fe_3_O_4_@polydopamine as recyclable nanocatalyst. Solid State Sci..

[59] Panigrahi S., Kundu S., Ghosh S., Nath S., Pal T. (2004). General method of synthesis for metal nanoparticles. J. Nanopart. Res..

[60] Lu T. (2021). Simple, reliable, and universal metrics of molecular planarity. J. Mol. Model..

[61] Sarkisov L., Bueno-Perez R., Sutharson M., Fairen-Jimenez D. (2020). Material Informatics with PoreBlazer v4.0 and the CSD MOF Database. Chem. Mater..

[62] Lee M.-T. (2021). Designing Highly Conductive Block Copolymer-Based Anion Exchange Membranes by Mesoscale Simulations. J. Phys. Chem. B.

[63] Lee M.-T. (2020). Designing Anion Exchange Membranes with Enhanced Hydroxide Ion Conductivity by Mesoscale Simulations. J. Phys. Chem. C.

